# Identification and Characterization of MicroRNAs in the Goat (*Capra hircus*) Rumen during Embryonic Development

**DOI:** 10.3389/fgene.2017.00163

**Published:** 2017-10-26

**Authors:** Tao Zhong, Jiangtao Hu, Ping Xiao, Siyuan Zhan, Linjie Wang, Jiazhong Guo, Li Li, Hongping Zhang, Lili Niu

**Affiliations:** Farm Animal Genetic Resources Exploration and Innovation Key Laboratory of Sichuan Province, College of Animal Science and Technology, Sichuan Agricultural University, Chengdu, China

**Keywords:** goat, rumen development, embyronic stage, microRNAs, RNA-sequencing

## Abstract

The rumen is an important digestive organ in ruminants. Numerous regulatory factors including microRNAs (miRNAs) are involved in embryonic organ development. In the present study, miRNAs expressed in the rumens of goats (*Capra hircus*) and their potential roles in the pathways involved in rumen development were identified using high-throughput sequencing. Histological morphology revealed a distinct difference in each layer of rumen during the period from embryonic day 60 (E60) to embryonic day 135 (E135). We determined the expression profiles of miRNAs in the goat rumen, and identified 423 known miRNAs and 559 potentially novel miRNAs in the E60 and E135 embryonic rumen, respectively. Bioinformatics analysis annotated the 42 differentially expressed miRNAs and the top 10 most highly expressed miRNAs of the two libraries to 48 and 38 gene ontology categories, as well as to 168 and 71 Kyoto Encyclopedia of Genes and Genomes pathways, respectively. The expression patterns of eight randomly selected miRNAs were validated by stem-loop quantitative reverse transcription PCR, suggesting that the sequencing data were reliable. We profiled the genome-wide expression of rumen-expressed miRNAs at different prenatal stages of rumen tissues, revealing that a subset of miRNAs might play important roles in the formation of the rumen layers. Taken together, these findings will aid the investigation of dominant rumen-related miRNA sets and help understand the genetic control of rumen development in goats.

## Introduction

The rumen, a specialist digestive organ of ruminants, plays a vital role in digestion metabolism. Its wall can be divided into four layers: the mucosa, submucosa, muscularis, and tunica adventitia. In goats (*Capra hircus*), rumen papillae appear to be separated by clear boundaries at embryonic day 64 (E64); by embryonic day 150 (E150), rumen papillae are fully developed and show signs of surface keratinization (Franco et al., 1992). To date, studies have focused on the effect of dietary composition and nutrition on rumen development (Coverdale et al., 2004; Gupta et al., 2016; Suarez-Mena et al., 2016). Naeem et al. (2012) also evaluated the expression of genes involved in rumen development, while several microRNAs (miRNAs) associated with development of the digestive tract have been reported (Hino et al., 2008; McKenna et al., 2010; Nguyen et al., 2010; Dalmasso et al., 2011; Kim et al., 2011; Ye et al., 2011; Chen et al., 2013). However, the role of miRNAs in the goat rumen during embryonic development has not been described.

MicroRNAs were originally identified as negative regulators of their target genes that binding to the sequences of 3′-untranslated region (3′-UTR) (Lim et al., 2005). They may also induce gene expression by targeting the promoter sequences of target genes (Place et al., 2008). Although miRNAs are encoded by only a small number of genes, they may regulate the expression of many mRNAs (Berezikov et al., 2005; Lewis et al., 2005). Increasing evidence has suggested that miRNAs are implicated in embryonic development (Wienholds et al., 2005), cell differentiation (Ivey and Srivastava, 2010), proliferation (Brennecke et al., 2003), apoptosis (Cimmino et al., 2005), and tumorigenesis (Yamanaka et al., 2012; Han et al., 2015).

Little genetic information is available about the development of the rumen in goats, and differences between miRNA profiles during the formation of rumen layers are also unknown. Therefore, in the present study, we used RNA sequencing technology to understand the complex regulation of rumen development at gestational day 60 (before rumen papillae formation) and gestational day 135 (after rumen papillae formation). Systematical analyses were carried out of known miRNAs, potentially novel miRNAs, differentially expressed miRNAs, and their target genes. We also observed the morphology of rumen tissues at different development stages (E60 and E135) to provide an insight into the mechanisms of miRNA regulation of rumen development.

## Materials and Methods

### Ethics Statement

All animal experiments were performed in strict accordance with the Regulations for the Administration of Affairs Concerning Experimental Animals (Ministry of Science and Technology, China, revised in June 2004) and given prior approval by the Institutional Animal Care and Use Committee at the College of Animal Science and Technology, Sichuan Agricultural University, Sichuan, China under permit No. DKY-B20110807. All animals were sacrificed humanely to minimize suffering.

### Rumen Collection and Total RNA Isolation

Pregnant does were provided by the Station of the Jianyang Da’er Goat Breeding Center (Sichuan, China). Six pregnant does at two embryonic stages (E60 and E135) were anesthetized by Zoletil (4 mg/kg body weight) for cesarean section. Rumen tissues were collected from the embryos (*n* = 3 per stage). The six rumen tissues were immediately frozen in liquid nitrogen, and then stored at -80°C until required for RNA isolation. Total RNA was isolated using TRIzol reagent (Invitrogen, Carlsbad, CA, United States). The purity, concentration, and quality of the RNA were determined by a Nanodrop 2000 spectrophotometer (Thermo Scientific Nanodrop, Wilmington, DE, United States), Qubit 3.0 Fluorometer (Life Technologies, Camarillo, CA, United States), and Agilent 2100 Bioanalyzer (Agilent Technologies, Palo Alto, CA, United States), respectively.

### Hematoxylin and Eosin Staining

The fixed rumen samples were dehydrated and embedded in paraffin. Sections (5-μm) were cut using a microtome (LeicaRM2016, Leica Microsystems, Germany) and placed on glass slides. The sections were stained with hematoxylin and eosin (H&E) and viewed using an Olympus BX51 microscope (Olympus, Tokyo, Japan) equipped with a Nikon DS-Fi1 camera (Nikon, Tokyo, Japan).

### Small RNA Library Construction and Sequencing

Approximately 1.5 μg of total RNA was used to generate small RNA libraries with TruSeq Small RNA Sample Prep Kits (Illumina, San Diego, CA, United States) according to the manufacturer’s protocol. Briefly, small RNAs were ligated to 3′ and 5′ RNA adapters. The ligated small RNAs were reverse transcribed and amplified by PCR. The PCR products were then purified by denaturing polyacrylamide gel electrophoresis (PAGE). After examining the concentration and insert size by a Qubit 3.0 Fluorometer (Life Technologies, Camarillo, CA, United States) and Agilent 2100 Bioanalyzer (Agilent Technologies, Palo Alto, CA, United States), the libraries were subjected to 50 bp single-end reads sequencing on an Illumina HiSeq 2500 platform (Illumina, San Diego, CA, United States).

### Sequencing Data Analysis and Identification of miRNAs

Raw reads in fastq format were filtered out the low-quality reads, reads containing poly-*N* (>10% unknown bases), and reads containing adapter using in-house scripts (fastq v1.1^1^) written in Perl. Then reads were trimmed and cleaned by removing the sequences smaller than 18 nt or longer than 30 nt. In addition, Q30 of the clean data were also calculated. The clean reads were aligned with Silva, GtRNAdb, Rfam, and Repbase databases, respectively, to filter rRNA, tRNA, snRNA, snoRNA, other ncRNA, and repeat sequences using the Bowtie v.1.1.0 software (Langmead et al., 2009). The derived reads were used to detect known goat miRNAs by aligned to the goat miRNA precursors present in the miRBase v21^2^ as described in our previous study (Guo et al., 2016a). Clear reads were also mapped to the goat reference genome (CHIR_1.0, NCBI) using the Bowtie aligner. The mapped reads were further denoted as known miRNAs under the following criteria: (i) aligned to the mature known miRNA precursors of other animal species in miRBase, (ii) raw count for known miRNAs was quantified and normalized using transcripts per million (TPM) mapped reads. Only those miRNAs, an overall expression level greater than one RPM in six libraries, were denoted as true known miRNAs. The unannotated reads which mismatched to any public databases or goat genome were further analyzed to identify novel miRNAs using Mirdeep2 v2.0.0.7 (Friedlander et al., 2012), with the default options (e.g., minimal free energy < -18 kcal/mol) as described in our previous study (Guo et al., 2016a).

### Differential Expression Analysis of miRNAs

To assess the differentially expressed miRNAs, the DESeq2 package in the statistics software R (Version 3.0.1) was used with standard parameters (Love et al., 2014). In order to avoid false positive results, the two criteria: only |log_2_ (Fold change)| ≥ 1 and the false-discovery rate (FDR) adjusted *p*-value ≤ 0.01 were used to determine the differentially expressed miRNAs.

### Target Prediction and Functional Annotation of Target Genes

TargetScan (Lewis et al., 2003), RNAhybrid (Kruger and Rehmsmeier, 2006), and miRanda (Betel et al., 2010) prediction tools were used to predict the target genes of differentially expressed miRNAs. To filter out the false positives, the predicted target genes were retained only when they were identified by all the three programs.

The predicted target gene candidates of differentially expressed miRNAs were mapped in NR (Deng et al., 2006), Swiss-Prot (Apweiler et al., 2004), gene ontology (GO) (The Gene Ontology Consortium et al., 2000), COG (Tatusov et al., 2000), Kyoto Encyclopedia of Genes and Genomes (KEGG) (Kanehisa et al., 2004), KOG (Koonin et al., 2004), and Pfam (Eddy, 1998) databases to obtain annotation information. GO enrichment analysis^3^ was performed using three ontologies (cellular component, molecular function, and biological process). To understand the biological functions of target genes, KEGG pathway analysis was performed.

### Validation of Differentially Expressed miRNAs

To validate the sequencing data, eight randomly selected miRNAs (including six known and two novel miRNAs) were validated by stem-loop quantitative real-time reverse transcription PCR (qRT-PCR). Total RNA (5 ng) was reverse transcribed into cDNA using the Mir-X^TM^ miRNA First-Strand Synthesis Kit (TaKaRa, Dalian, China) according to the user’s manual. To detect the expression patterns of target genes of differentially expressed miRNAs, cDNA was synthesized using the PrimeScript RT Reagent Kit (TaKaRa, Dalian, China). The cDNA products were diluted 1:5 (v/v) with EASY Dilution (TaKaRa, Dalian, China) and stored at -20°C. Primers used in this study are shown in Supplementary Table S8.

Quantitative real-time reverse transcription PCR was performed using a CFX Connect Real-Time PCR Detection System (Bio-Rad Laboratories, Singapore) in a volume of 10 μL including 5 μL of SYBR Green Real Time PCR Master Mix (TaKaRa, Dalian, China), 0.2 μL each of forward and reverse primer, 3.8 μL of RNase-Free dH_2_O, and 0.8 μL of cDNA. U6 snRNA was used as the reference for miRNA and target gene candidates (Guo et al., 2016a). All reactions were performed in triplicate, and the relative expression level was normalized by 2^-ΔΔC_T_^ method.

## Results

### Histological Observation of the Embryonic Goat Rumen

**FIGURE 1:**
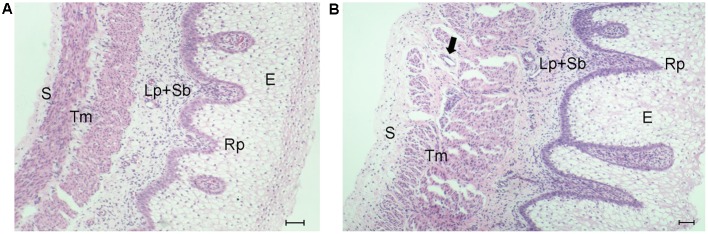
Histological observation of embryonic goat rumen tissues. The wall is composed of four layers: epithelium (E), lamina propria and submucosa (Lp + Sb), tunica muscularis (Tm), and serosa (S). **(A)** Photomicrograph of rumen wall at E60. **(B)** Photomicrograph of rumen wall at E135. The arrow indicates the blood vessel. Bar, 100 μm.

To observe the differences between E60 and E135 goat rumens, H&E-stained sections were prepared. As shown in **Figure 1**, each layer of E60 rumen was thinner than that those of the E135 rumen. At E135, rumen papillae were more developed than at E60, appearing as evaginations of the stratum basale toward the stratum granulosum. Moreover, cells in the lamina propria were more closely packed, the diameters of muscle fibers were increased, and the spaces between muscle fibers were wider. Finally, more numerous blood vessels were found in the tunica muscularis.

### Overview of Sequencing Data

To obtain a transcriptome of the caprine rumen at different developmental stages, two libraries were constructed from goats at E60 and E135. An average of 18,810,062 and 21,162,680 raw reads was obtained from E60 and E135, respectively (**Table 1**). After removing low-quality sequences and adapters, an average of 15,500,932 and 19,252,745 clean reads, respectively, was retained (**Table 1**). The rRNA, scRNA, snRNA, snoRNA, tRNA, and repeat sequences were filtered from clean reads to obtain an average of 13,630,047 and 17,583,220 reads, respectively, which were mapped to the goat genome (CHIR_1.0, NCBI) using the miRDeep2 program (Supplementary Table S1). The reads between 18 and 30 nt were BLASTed against the miRBase, which identified 423 known miRNAs and 559 novel miRNAs (Supplementary Table S2).

**Table 1:** Summary of reads from raw data and clean reads for microRNAs (miRNAs) sequencing in goat rumen.

Item	E60 stage			E135 stage		
	S01	S02	S03	S04	S05	S06
Total reads number	19794964	18233301	18401922	18944864	21145362	23397815
Filter low-quality reads	0	0	0	0	0	0
Filter having ‘*N*’ reads	8235	7662	7437	15037	16444	18360
Length < 18	316188	800029	315609	359100	247396	603251
Length > 30	3423933	3586672	1461627	1120443	1099306	2250469
Clean reads	16046608	13838938	16617249	17450284	19782216	20525735
Q30 (%)	92.61	92.05	92.93	93.85	93.90	93.56

*S01, S02, and S03 indicate the three biological samples at E60. S04, S05, and S06 indicate the three biological samples at E135*.

### Expression Analysis of miRNAs in the Embryonic Goat Rumen

**FIGURE 2:**
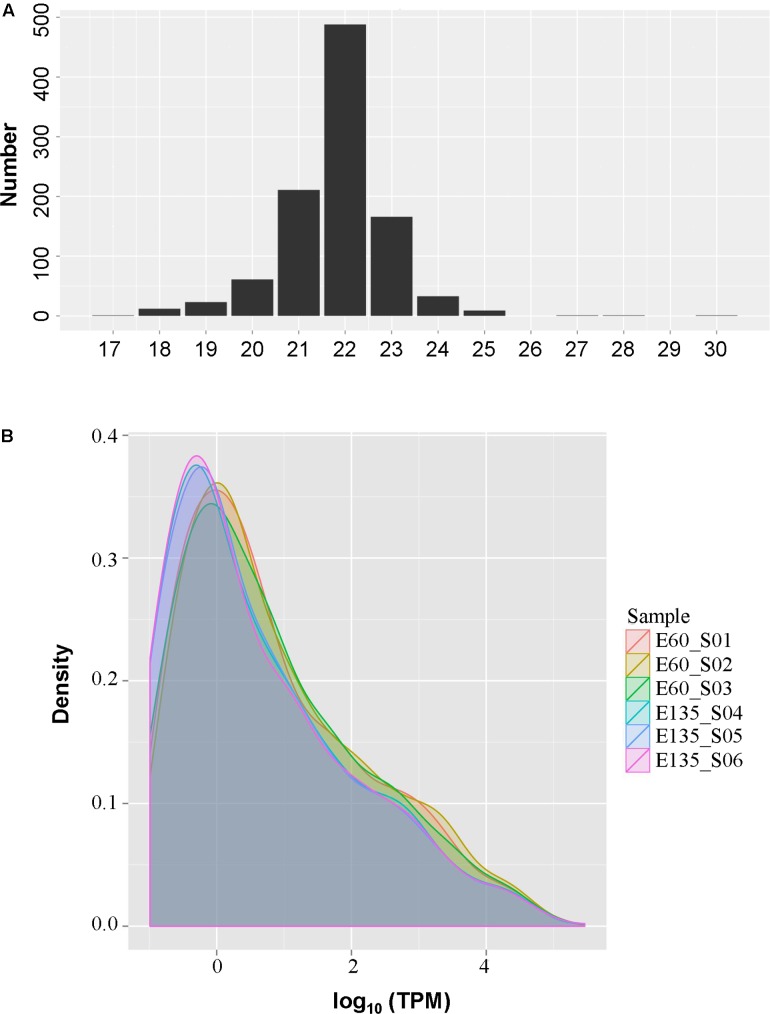
The size **(A)** and expression density **(B)** distributions of the microRNAs (miRNAs) in goat rumen tissues.

As shown in **Figure 2A**, most miRNAs were 22 nt in length, followed by 21 and 23 nt, indicating good sequencing quality. Expression density distributions were analyzed according to the normalized reads. As shown in **Figure 2B**, the expression levels were highly consistent among the six tested samples and between the different embryonic periods (**Supplementary Figure S1**). Differential expression analysis found that 42 miRNAs were differentially expressed, of which 22 miRNAs were up-regulated and 20 were down-regulated: 27 of the miRNAs were known while 15 were novel (**Table 2**). However, sample size is an important point for the determination of DE miRNAs, less abundant miRNAs might be hidden in a small sample size.

**Table 2:** List of the differentially expressed miRNAs identified at E60 and E135 rumen tissues.

miRNAs	E60 rumen			E135 rumen			FDR	log_2_FC	Regulated
	S01	S02	S03	S04	S05	S06			
chi-let-7a-5p	13720.667	11681.573	11654.581	14407.907	15088.169	16068.102	0.0014	1.1325	Up
chi-let-7b-5p	3276.443	3529.129	2905.827	6073.946	6342.372	6277.083	0.0001	1.7827	Up
chi-miR-10a-5p	35283.422	51769.951	22437.156	45157.094	48263.762	47406.678	0.0000	1.2045	Up
chi-miR-130b-5p	278.300	209.185	284.005	46.547	46.456	48.809	0.0022	-1.6095	Down
chi-miR-137	21.237	9.224	17.199	2.089	1.231	1.451	0.0014	-2.4899	Down
chi-miR-143-3p	129549.751	108999.296	131907.860	289042.056	244086.093	254802.244	0.0000	1.9254	Up
chi-miR-145-5p	23595.939	6821.073	16046.882	28255.511	27051.762	23796.398	0.0000	1.5921	Up
chi-miR-147-3p	0.972	2.141	1.005	4.759	4.512	5.596	0.0001	2.6986	Up
chi-miR-150	21.931	19.107	26.245	37.841	42.149	37.306	0.0044	1.6389	Up
chi-miR-182	271.360	251.351	469.507	666.513	719.920	715.658	0.0034	1.9218	Up
chi-miR-195-3p	0.972	1.482	1.340	10.447	10.255	11.295	0.0000	3.9190	Up
chi-miR-22-3p	7.634	23.389	13.402	31.225	29.228	33.472	0.0007	1.9312	Up
chi-miR-23a	1109.590	1772.636	954.202	2081.952	2340.560	2306.976	0.0036	1.6534	Up
chi-miR-24-3p	1286.981	1776.753	1358.264	2650.496	2409.886	2473.610	0.0050	1.6109	Up
chi-miR-27a-3p	405.860	585.388	377.705	1083.576	874.877	859.287	0.0024	1.8852	Up
chi-miR-27b-3p	22088.401	22551.772	26534.396	35528.994	36220.178	33742.134	0.0000	1.4067	Up
chi-miR-323a-3p	477.482	503.032	314.605	55.949	54.353	72.851	0.0006	-1.9872	Down
chi-miR-34a	24.152	29.154	15.300	58.038	63.378	55.959	0.0000	2.2096	Up
chi-miR-34c-5p	109.377	107.063	118.717	16.483	21.844	17.202	0.0007	-1.7559	Down
chi-miR-412-5p	550.076	683.557	436.561	80.325	67.377	99.794	0.0014	-1.9168	Down
chi-miR-412-3p	3.470	4.447	1.787	0.116	0.103	0.311	0.0010	-3.3652	Down
chi-miR-485-3p	68.013	101.133	64.663	17.063	11.076	14.404	0.0038	-1.6147	Down
chi-miR-497-5p	2.498	3.130	3.909	19.037	18.049	19.379	0.0000	3.4072	Up
chi-miR-504	76.480	107.063	122.514	19.269	14.973	15.752	0.0006	-1.7697	Down
chi-miR-592	12.492	4.777	14.742	0.580	0.513	0.104	0.0006	-3.9023	Down
chi-miR-708-3p	142.828	141.488	142.617	237.725	253.715	248.605	0.0059	1.6309	Up
chi-miR-96	13.464	8.400	17.869	34.939	32.714	27.151	0.0005	2.0909	Up
Novel_022293.1_14794	4.442	4.283	6.254	0.348	0.308	0.518	0.0002	-2.8382	Down
Novel_022294.1_86626	3.886	2.635	5.361	0.348	0.000	0.415	0.0001	-3.1294	Down
Novel_022297.1_202845	4.025	2.800	5.696	0.232	0.103	0.311	0.0000	-3.4453	Down
Novel_022299.1_294806	2.221	1.482	3.015	0.348	0.308	0.207	0.0047	-2.1217	Down
Novel_022302.1_372634	0.694	0.329	0.223	4.643	6.461	6.839	0.0000	4.6678	Up
Novel_022302.1_396688	3.748	2.306	5.361	0.232	0.000	0.207	0.0001	-3.8644	Down
Novel_022308.1_581193	10.965	16.471	7.818	1.393	1.026	1.451	0.0014	-2.3461	Down
Novel_022308.1_587455	10613.564	7692.566	8985.963	14649.462	12688.538	14613.677	0.0001	1.4534	Up
Novel_022313.1_710968	3.748	2.306	5.361	0.232	0.000	0.207	0.0001	-3.8644	Down
Novel_022314.1_720489	3.886	2.635	5.361	0.348	0.000	0.415	0.0001	-3.1294	Down
Novel_022315.1_738871	1.804	1.812	2.122	5.340	6.358	5.596	0.0007	2.4297	Up
Novel_022320.1_827014	0.555	1.318	1.005	2.786	2.871	3.005	0.0010	2.4379	Up
Novel_022322.1_857850	50.802	74.285	30.489	0.464	0.103	0.000	0.0000	-7.2415	Down
Novel_022322.1_874240	4.164	2.471	6.366	0.232	0.000	0.104	0.0002	-4.4328	Down
Novel_022322.1_879864	4.164	2.471	6.366	0.232	0.000	0.104	0.0002	-4.4328	Down

We also focused on miRNAs with high TPM in each library. The reads of the top 10 most highly expressed miRNAs accounted for 50.6 and 63.9% of the total average reads, respectively, in each library. A total of 11 miRNAs are listed in the top 10 of the two libraries, of which 9 (except miR-7-5p and miR-145-5p) are found in both libraries (**Table 3**).

**Table 3:** Top 10 most highly expressed miRNAs of two libraries.

miRNAs	E60 stage			E135 stage		
	S01 TPM	S02 TPM	S03 TPM	S04 TPM	S05 TPM	S06 TPM
miR-143-3p	129549.75	108999.30	131907.86	289042.06	244086.09	254802.24
let-7i-5p	89275.67	59837.40	80051.92	49247.64	57126.98	55985.45
miR-148a-3p	49231.45	63525.48	60970.78	54450.2	58719.63	75040.85
let-7f-5p	48908.45	38014.15	49118.86	41210.49	50400.24	45654.01
miR-21-5p	34218.80	42380.02	39907.41	37213.73	39477.66	36745.39
miR-26a-5p	42075.88	29769.31	37773.64	38567.65	43951.01	39828.85
miR-10a-5p	35283.42	51769.95	22437.16	45157.09	48263.76	47406.68
let-7g-5p	39740.11	27358.58	33315.22	25805.71	28717.01	26394.67
miR-7-5p	40366.80	12551.75	36865.33	3029.37	3103.24	3731.87
miR-27b-3p	22088.40	22551.77	26534.4	35528.99	36220.18	33742.13
miR-145-5p	23595.94	6821.07	16046.88	28255.51	27051.76	23796.4

### GO Enrichment Analysis and KEGG Pathway Analysis of Target Genes

Target gene prediction found that 223 genes were predicted to be targeted by 18 differentially expressed miRNAs, while 65 genes were predicted to be targeted by 6 of the top 10 most highly expressed miRNAs (Supplementary Tables S3, S4). Supplementary Table S5 shows the annotation of target genes for all miRNAs.

**FIGURE 3:**
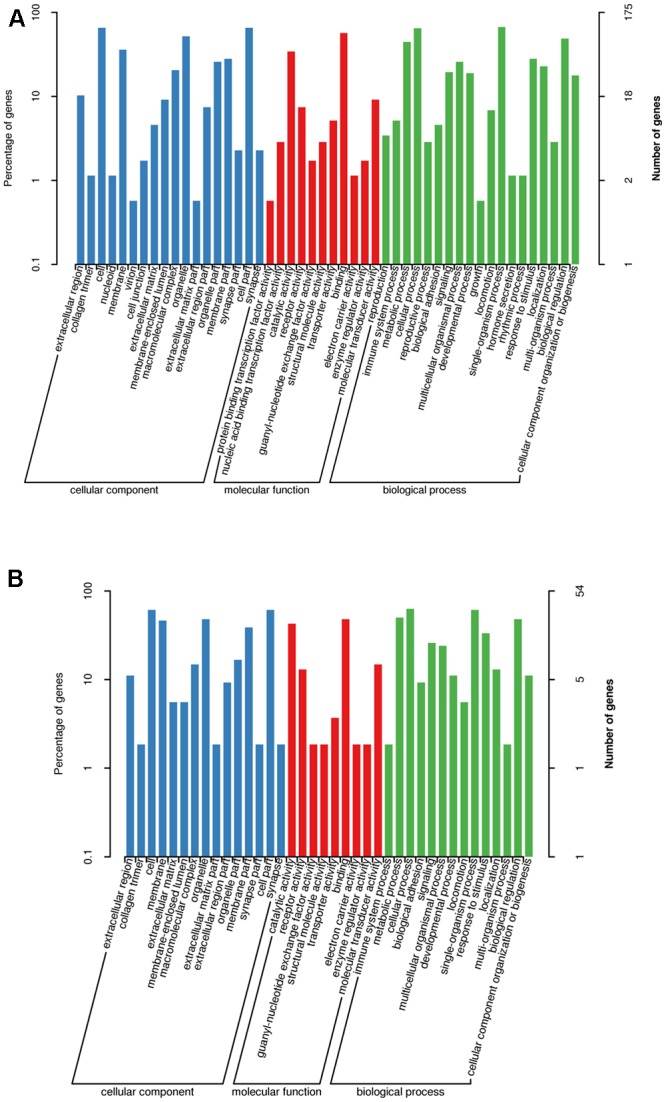
Partial gene ontology classification for predicted miRNAs. **(A)** Gene ontology classification for differentially expressed miRNAs. **(B)** Gene ontology classification for top 10 highly expressed miRNAs.

Gene ontology enrichment analysis of these putative target genes revealed that 175 and 54 target genes of differentially expressed and the top 10 most highly expressed miRNAs, respectively, were annotated. As shown in **Figure 3**, the most enriched GO categories of differentially expressed miRNAs were consistent with those of the top 10 most highly expressed miRNAs. The analysis of cellular component showed that most genes were involved in cell and cell part categories. The analysis of molecular function showed that most genes were clustered into binding and catalytic activities. Moreover, the analysis of biological process showed that most genes were related to single organism and cellular processes.

Kyoto Encyclopedia of Genes and Genomes pathway annotation showed that 168 and 71 biological functions were annotated by target genes of differentially expressed and the top 10 most highly expressed miRNAs, respectively (Supplementary Tables S6, S7). The enriched pathways for target genes of differentially expressed miRNAs mainly included the Ras signaling pathway, the phosphoinositide 3-kinase (PI3K)-Akt signaling pathway, focal adhesion, and regulation of the actin cytoskeleton. KEGG pathway analysis based on predicted targets revealed that the top 10 most highly expressed miRNAs were involved in the mitogen-activated protein kinase (MAPK) signaling pathway, the Ras signaling pathway, the PI3K-Akt signaling pathway, and olfactory transduction.

### Quantitative Real-Time-PCR Validation of the Differentially Expressed miRNAs

**FIGURE 4:**
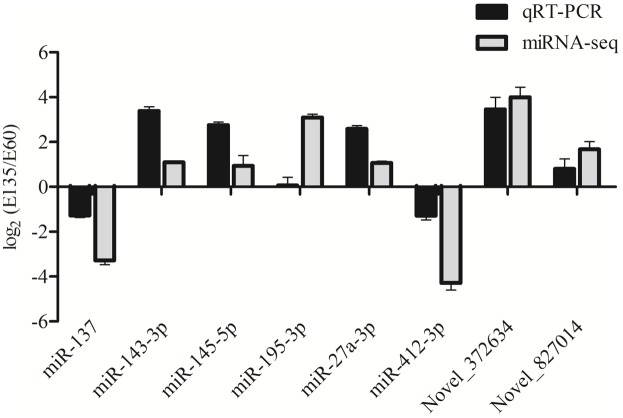
Validation of differentially expressed miRNAs by stem-loop qRT-PCR.

Eight miRNAs, including six known and two novel miRNAs, were randomly selected to validate the reliability of the sequencing data. As shown in **Figure 4**, relative expression changes of miRNAs were represented using log_2_ (E135/E60). All selected miRNAs showed expression changes that were consistent with the deep sequencing results, suggesting that the sequencing data were credible.

## Discussion

Histological analysis revealed that the goat E60 rumen is composed of four layers, and that the rumen mainly undergoes a process of tissue growth during the period from E60 to E135. Rudimentary pillars have previously been observed in the E46 rumen, whose wall lacked a complete structure (Garcia et al., 2012). This indicates that samples earlier than E46 should be used to identify miRNAs with important roles in rumen development.

Embryonic rumen development involves the formation and differentiation of the rumen wall, and differentiation of the epithelial layer. The molecular mechanism for this complex biological process has not been reported yet. However, numerous studies have focused on the development and regulatory mechanisms of intestinal epithelial cells, and Wnt and Notch signaling pathways were identified as determining factors for cell fate (Nakamura et al., 2007). In this study, differentially expressed miRNAs such as miR-182 (Kim et al., 2012), miR-23a (Wang et al., 2012), and miR-195-3p (Chen et al., 2011) were reported to mainly take part in the regulation of cell proliferation, differentiation, and apoptosis. Moreover, miR-34a was shown to promote renal senescence and regulate neural stem cell differentiation (Aranha et al., 2011; Bai et al., 2011), while miR-592 regulates neuronal apoptosis through p75^NTR^ (Irmady et al., 2014). These findings suggest that differentially expressed miRNAs are involved in cell differentiation and apoptosis, mitochondrial metabolism and neuronal development of the goat rumen. Moreover, the top 10 most highly expressed miRNAs, such as miR-26a-5p (Guo et al., 2016b) and miR-27b-3p (Tao et al., 2015), were also implicated in cell proliferation. Interestingly, let-7i-5p, let-7f-5p, and miR-21-5p are highly expressed in the porcine ovary and testis, suggesting that they are associated with secretory function (Li et al., 2011). miR-143 and miR-145 are co-expressed miRNAs that are not expressed in colonic epithelial tissue (Kent et al., 2014). Deep sequencing results revealed that they were highly expressed in the goat rumen, although the expression of miR-145 was significantly lower than that of miR-143 in both E60 and E135, which may be caused by post-transcriptional regulation. The expression of the conserved miRNAs in goat rumen revealed that the top 10 abundant miRNAs might act important roles in the development or physiology of rumen layers. These miRNAs could be studied as the preferred candidate miRNAs for regulation analysis of rumen development in ruminants. However, it is also crucial to point that DE miRNAs associated with rumen development could be limited by the small sample size in our study. Therefore, the evaluation of DE miRNAs in goat rumen with larger sample size might produce more reliable and compatible results.

Functional enrichment analysis revealed that the differentially expressed and top 10 most highly expressed miRNAs shared similar functions (Supplementary Table S5 and **Figure 3**). GO annotation analysis revealed that a large proportion of the genes of miRNA targets in goat rumen were involved in the (extra)cellular structures and cytoskeleton, which had binding and catalytic functions. Consistently, no specifically expressed miRNAs were found between E60 and E135. The most enriched pathways, including MAPK, Ras, and PI3K-Akt signaling pathways, are implicated in gastric cell proliferation, intestinal epithelial cell growth, and intestinal epithelial cell proliferation, respectively (Osaki et al., 2011; Waseem et al., 2014; Depeille et al., 2015). In addition, KEGG analysis also showed that approximately 24% pathways of the top 10 miRNAs were committed to internal secretion, cancer, leukemia, and virus infection diseases (Supplementary Table S7). These results indicated that some miRNAs might be involved in cell proliferation, apoptosis, and phagocytosis. Further research is required to demonstrate the relationship between miRNAs and target mRNAs transcripts and their pathways in the development of the goat rumen.

## Conclusion

This study identified miRNAs of the E60 and E135 goat rumen and evaluated expression of these miRNAs at the two time points. A total of 423 known miRNAs and 559 novel miRNAs were identified. Analysis of 42 differentially expressed and the 11 top most highly expressed miRNAs revealed that most of their target genes are involved in cellular activities such as proliferation, differentiation, and apoptosis, which is consistent with histological observations. To identify miRNAs that play essential roles in early rumen development, samples before E46 should be examined. However, our current results provide useful information for further research, which will contribute to the elucidation of the molecular mechanisms involved in rumen development.

## Author Contributions

TZ conceived and designed the experiments. JH and PX performed the experiments. JH and SZ analyzed the data. LW, JG, LL, and HZ participated in sample collection. HZ, LN, and TZ contributed reagents/materials. JH and TZ wrote the manuscript.

## Conflict of Interest Statement

The authors declare that the research was conducted in the absence of any commercial or financial relationships that could be construed as a potential conflict of interest. The reviewer RS and handling Editor declared their shared affiliation.

## Footnotes

1 https://github.com/ZHONGTAO2017/filter/blob/data-filter/.gitattributes

2 http://www.mirbase.org

3 http://www.geneontology.org/

## Supplementary Material

FIGURE S1 Box plot of the expression levels among the six tested rumen samples.
