# Supplementary figures and images for: Identification and Characterization of MicroRNAs in the Goat (Capra hircus) Rumen during Embryonic Development
Source: Front Genet. 2017 Oct 26;8:163. doi: 10.3389/fgene.2017.00163 (PMC5662549; doi:10.3389/fgene.2017.00163)

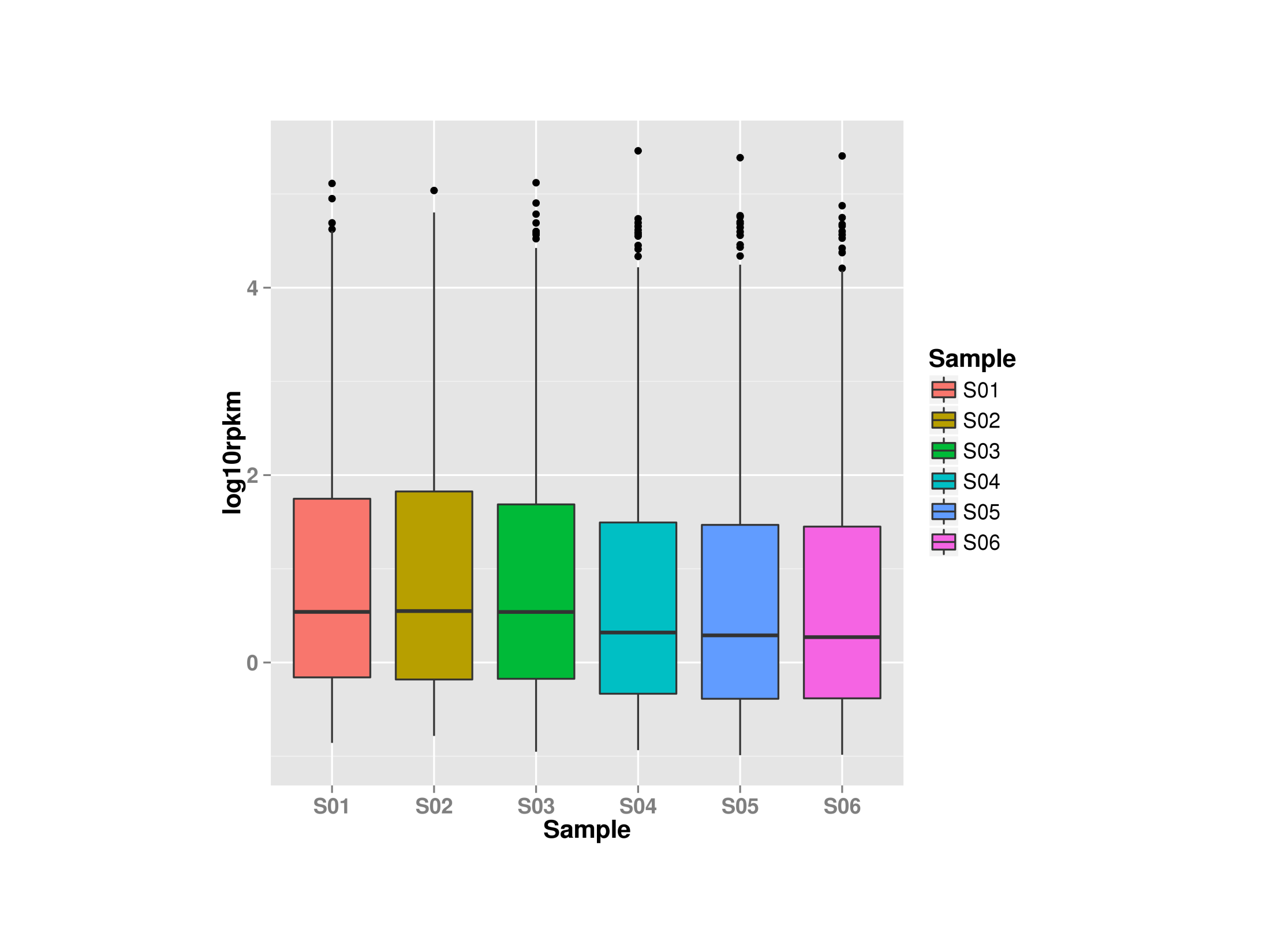

Supplement: FIGURE S1 — Box plot of the expression levels among the six tested rumen samples. [file Image_1.TIFF]
